# *cis*-Bis(2,2′-bipyrimidine-κ^2^ *N* ^1^,*N* ^1′^)dibromidomanganese(II) nitro­methane monosolvate

**DOI:** 10.1107/S1600536811010075

**Published:** 2011-03-23

**Authors:** Kwang Ha

**Affiliations:** a School of Applied Chemical Engineering, The Research Institute of Catalysis, Chonnam National University, Gwangju 500-757, Republic of Korea

## Abstract

The asymmetric unit of the title compound, [MnBr_2_(C_8_H_6_N_4_)_2_]·CH_3_NO_2_, contains one half of a neutral Mn^II^ complex and one half of a nitro­methane solvent mol­ecule, the complete mol­ecules being generated by the application of twofold symmetry. In the complex, the Mn^II^ ion has a distorted *cis*-Br_2_N_4_ octa­hedral coordination geometry defined by four N atoms of the two chelating 2,2′-bipyrimidine ligands and two Br^−^ ions. There are intra- and inter­molecular C—H⋯Br and C—H⋯N contacts.

## Related literature

For the crystal structures of mononuclear 2,2′-bipyrimidine Mn^II^ complexes, see: Hong *et al.* (1996 ▶); Smith *et al.* (2001 ▶). 
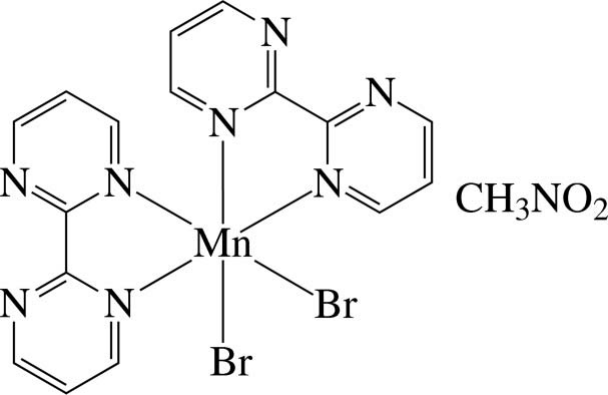


## Experimental

### 

#### Crystal data

[MnBr_2_(C_8_H_6_N_4_)_2_]·CH_3_NO_2_
*M* *_r_* = 592.14
Orthorhombic, 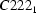

*a* = 8.3876 (4) Å
*b* = 12.1772 (6) Å
*c* = 20.7479 (11) Å
*V* = 2119.14 (18) Å^3^
*Z* = 4
Mo *K*α radiation
μ = 4.43 mm^−1^
*T* = 200 K
0.17 × 0.10 × 0.07 mm

#### Data collection

Bruker SMART 1000 CCD diffractometer
Absorption correction: multi-scan (*SADABS*; Bruker, 2000 ▶) *T*_min_ = 0.825, *T*_max_ = 1.000
7872 measured reflections
2608 independent reflections
1818 reflections with *I* > 2σ(*I*)
*R*_int_ = 0.069

#### Refinement

*R*[*F* ^2^ > 2σ(*F* ^2^)] = 0.043
*wR*(*F* ^2^) = 0.078
*S* = 1.02
2608 reflections
143 parameters
H-atom parameters constrained
Δρ_max_ = 0.87 e Å^−3^
Δρ_min_ = −0.86 e Å^−3^
Absolute structure: Flack (1983 ▶), 1121 Friedel pairs
Flack parameter: −0.003 (16)

### 

Data collection: *SMART* (Bruker, 2000 ▶); cell refinement: *SAINT* (Bruker, 2000 ▶); data reduction: *SAINT*; program(s) used to solve structure: *SHELXS97* (Sheldrick, 2008 ▶); program(s) used to refine structure: *SHELXL97* (Sheldrick, 2008 ▶); molecular graphics: *ORTEP-3* (Farrugia, 1997 ▶) and *PLATON* (Spek, 2009 ▶); software used to prepare material for publication: *SHELXL97*.

## Supplementary Material

Crystal structure: contains datablocks global, I. DOI: 10.1107/S1600536811010075/tk2729sup1.cif

Structure factors: contains datablocks I. DOI: 10.1107/S1600536811010075/tk2729Isup2.hkl

Additional supplementary materials:  crystallographic information; 3D view; checkCIF report

## Figures and Tables

**Table:** 

Mn1—Br1	2.6140 (9)
Mn1—N1	2.292 (5)
Mn1—N3	2.306 (5)

**Table:** 

N1^i^—Mn1—N1	158.0 (2)
N3—Mn1—N3^i^	89.5 (2)
Br1—Mn1—Br1^i^	98.66 (5)

Symmetry code: (i) 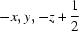.

**Table 2:** Hydrogen-bond geometry (Å, °)

*D*—H⋯*A*	*D*—H	H⋯*A*	*D*⋯*A*	*D*—H⋯*A*
C1—H1⋯Br1	0.95	2.87	3.542 (6)	129
C2—H2⋯Br1^ii^	0.95	2.91	3.802 (6)	156
C6—H6⋯Br1^iii^	0.95	2.86	3.752 (5)	157
C9—H9*C*⋯N2^iv^	0.98	2.57	3.430 (5)	147

Symmetry codes: (ii) 
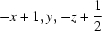
; (iii) 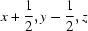; (iv) 
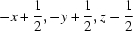
.

**Fig. 1.**
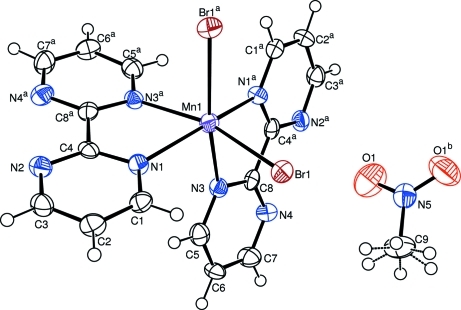
Molecular structure of the title compound, with displacement ellipsoids drawn at the 50% probability level for non-H atoms [Symmetry codes: (a) -x, y, 1/2 - z, (b) x, 1 - y, -z]. H atoms are shown as small circles of arbitrary radius and the bonds of the disordered methyl group are shown with dashed lines.

**Fig. 2.**
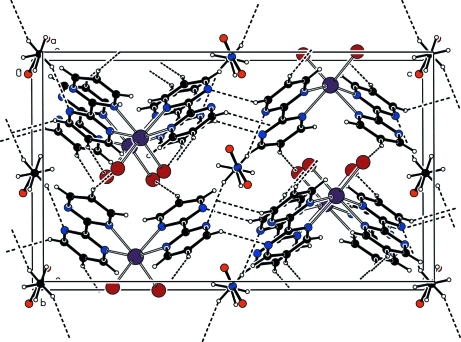
View of the unit-cell contents of the title compound. Hydrogen-bond interactions are drawn with dashed lines.

## supplementary crystallographic information

### Comment

Mononuclear Mn^II^ complexes of 2,2'-bipyrimidine ligand have been investigated previously (Hong *et al.*, 1996; Smith *et al.*, 2001). The asymmetric unit of the title compound, [MnBr_2_(bpym)_2_].CH_3_NO_2_ (where bpym is 2,2'-bipyrimidine; C_8_H_6_N_4_), contains half of a neutral Mn(II) complex and half of a nitromethane solvent molecule (Fig. 1). The complex is disposed about a twofold rotation axis running in the [010] direction passing through the Mn1 atom with the special position at (0, *y*, 1/4) (Wyckoff letter b). The solvent molecule is also located on a twofold rotation axis, but the axis is running along the [100] direction passing through the N5 and C9 atoms, which lie on the special positions of (*x*, 1/2, 0) (Wyckoff letter a). Because of the symmetry of the twofold rotation, the methyl group of the nitromethane molecule was modelled as disordered over two sites.

In the complex, the Mn^II^ ion is six-coordinated in a distorted octahedral environment defined by four N atoms of the two chelating 2,2'-bipyrimidine ligands and two Br^-^ anions, Table 1, which define a *cis*-Br_2_N_4_ donor set. The main contributions to the distortion are the tight N—Mn—N chelate angle and the Br—Br repulsion (N1—Mn1—N3*^a^* = 71.1 (2) ° and Br1—Mn1—Br1*^a^* = 98.66 (5) °; symmetry code a: -*x*, *y*, 1/2 - *z*), which result in non-linear *trans* axes (<N3—Mn1—Br1*^a^* = 163.6 (1) ° and <N1—Mn1—N1*^a^* = 158.0 (2) °). The Mn—N bond lengths are almost equivalent (Table 1) and the dihedral angle between the least-squares planes of the two bpym ligands is 73.05 (7) °.

In the crystal structure, the complexes are stacked in columns along the *a* axis. When viewed down the *c* axis, the successive complexes are stacked in the opposite direction. In the columns, several inter- and intramolecular π-π interactions between adjacent pyrimidine rings are present. The shortest distance between *Cg*1 (the centroid of ring N1—C4) and *Cg*2^i^ (ring N3—C8, symmetry code i: 1/2 - *x*, 1/2 + *y*, 1/2 - *z*) is 4.411 (3) Å, and the dihedral angle between the ring planes is 4.7 (3) °. Moreover, there are intra- and inter-molecular C—H···Br and C—H···N contacts with d(C···Br) = 3.542 (6)–3.802 (6) Å, and d(C···N) = 3.430 (5) Å (Fig. 2, Table 2).

### Experimental

To a solution of MnBr_2_.4H_2_O (0.2867 g, 1.000 mmol) in EtOH (30 ml) was added 2,2'-bipyrimidine (0.1584 g, 1.002 mmol) followed by stirring for 3 h. The precipitate was separated by filtration, washed with EtOH and dried at 323 K to give a yellow powder (0.3441 g). Crystals suitable for X-ray analysis were obtained by slow evaporation from a CH_3_NO_2_ solution.

### Refinement

H atoms were positioned geometrically and allowed to ride on their respective parent atoms [C—H = 0.95 Å (CH) or 0.98 Å (CH_3_), and with *U*_iso_(H) = 1.2*U*_eq_(C) or 1.5*U*_eq_(methyl C)]. The H atoms of the methyl group of the nitromethane solvent molecule were modelled as disordered over two sites rotated by 60° from one another, with an occupancy ratio of 0.5:0.5.

### Crystal data

**Table:**

[MnBr_2_(C_8_H_6_N_4_)_2_]·CH_3_NO_2_	*F*(000) = 1164
*M**_r_* = 592.14	*D*_x_ = 1.856 Mg m^−^^3^
Orthorhombic, *C*222_1_	Mo *K*α radiation, λ = 0.71073 Å
Hall symbol: C 2c 2	Cell parameters from 2401 reflections
*a* = 8.3876 (4) Å	θ = 3.0–25.5°
*b* = 12.1772 (6) Å	µ = 4.43 mm^−^^1^
*c* = 20.7479 (11) Å	*T* = 200 K
*V* = 2119.14 (18) Å^3^	Block, yellow
*Z* = 4	0.17 × 0.10 × 0.07 mm

### Data collection

**Table:**

Bruker SMART 1000 CCD diffractometer	2608 independent reflections
Radiation source: fine-focus sealed tube	1818 reflections with *I* > 2σ(*I*)
graphite	*R*_int_ = 0.069
φ and ω scans	θ_max_ = 28.3°, θ_min_ = 2.0°
Absorption correction: multi-scan (*SADABS*; Bruker, 2000)	*h* = −10→11
*T*_min_ = 0.825, *T*_max_ = 1.000	*k* = −16→15
7872 measured reflections	*l* = −24→27

### Refinement

**Table:**

Refinement on *F*^2^	Secondary atom site location: difference Fourier map
Least-squares matrix: full	Hydrogen site location: inferred from neighbouring sites
*R*[*F*^2^ > 2σ(*F*^2^)] = 0.043	H-atom parameters constrained
*wR*(*F*^2^) = 0.078	*w* = 1/[σ^2^(*F*_o_^2^) + (0.010*P*)^2^] where *P* = (*F*_o_^2^ + 2*F*_c_^2^)/3
*S* = 1.01	(Δ/σ)_max_ < 0.001
2608 reflections	Δρ_max_ = 0.87 e Å^−^^3^
143 parameters	Δρ_min_ = −0.86 e Å^−^^3^
0 restraints	Absolute structure: Flack (1983), 1121 Friedel pairs
Primary atom site location: structure-invariant direct methods	Flack parameter: −0.003 (16)

### Special details

**Table:**

Geometry. All e.s.d.'s (except the e.s.d. in the dihedral angle between two l.s. planes) are estimated using the full covariance matrix. The cell e.s.d.'s are taken into account individually in the estimation of e.s.d.'s in distances, angles and torsion angles; correlations between e.s.d.'s in cell parameters are only used when they are defined by crystal symmetry. An approximate (isotropic) treatment of cell e.s.d.'s is used for estimating e.s.d.'s involving l.s. planes.
Refinement. Refinement of *F*^2^ against ALL reflections. The weighted *R*-factor *wR* and goodness of fit *S* are based on *F*^2^, conventional *R*-factors *R* are based on *F*, with *F* set to zero for negative *F*^2^. The threshold expression of *F*^2^ > σ(*F*^2^) is used only for calculating *R*-factors(gt) *etc*. and is not relevant to the choice of reflections for refinement. *R*-factors based on *F*^2^ are statistically about twice as large as those based on *F*, and *R*- factors based on ALL data will be even larger.

### Fractional atomic coordinates and isotropic or equivalent isotropic displacement parameters (Å^2^)

**Table:**

	*x*	*y*	*z*	*U*_iso_*/*U*_eq_	Occ. (<1)
Br1	0.17577 (6)	0.51358 (4)	0.18610 (3)	0.03361 (16)	
Mn1	0.0000	0.37369 (10)	0.2500	0.0262 (3)	
N1	0.1984 (5)	0.3378 (3)	0.3230 (2)	0.0258 (11)	
N2	0.2612 (6)	0.2496 (4)	0.4234 (2)	0.0306 (12)	
N3	0.0862 (5)	0.2392 (4)	0.1799 (3)	0.0261 (10)	
N4	0.0382 (6)	0.1601 (4)	0.0767 (2)	0.0341 (13)	
C1	0.3439 (6)	0.3827 (4)	0.3225 (3)	0.0288 (12)	
H1	0.3728	0.4299	0.2880	0.035*	
C2	0.4533 (6)	0.3625 (5)	0.3707 (3)	0.0323 (15)	
H2	0.5572	0.3935	0.3694	0.039*	
C3	0.4059 (7)	0.2952 (5)	0.4210 (3)	0.0331 (16)	
H3	0.4789	0.2809	0.4550	0.040*	
C4	0.1658 (7)	0.2722 (4)	0.3736 (3)	0.0246 (12)	
C5	0.2306 (6)	0.1925 (4)	0.1809 (3)	0.0324 (14)	
H5	0.2971	0.2028	0.2175	0.039*	
C6	0.2853 (6)	0.1295 (5)	0.1300 (3)	0.0332 (15)	
H6	0.3876	0.0960	0.1308	0.040*	
C7	0.1849 (8)	0.1177 (5)	0.0783 (3)	0.0373 (14)	
H7	0.2214	0.0775	0.0420	0.045*	
C8	−0.0027 (7)	0.2208 (4)	0.1271 (3)	0.0255 (12)	
O1	−0.0029 (7)	0.4220 (4)	0.0230 (2)	0.0712 (16)	
N5	0.0678 (8)	0.5000	0.0000	0.0372 (18)	
C9	0.2426 (8)	0.5000	0.0000	0.046 (3)	
H9A	0.2815	0.4843	0.0436	0.069*	0.50
H9B	0.2815	0.5721	−0.0138	0.069*	0.50
H9C	0.2815	0.4435	−0.0297	0.069*	0.50

### Atomic displacement parameters (Å^2^)

**Table:**

	*U*^11^	*U*^22^	*U*^33^	*U*^12^	*U*^13^	*U*^23^
Br1	0.0310 (3)	0.0361 (3)	0.0337 (3)	−0.0004 (3)	0.0036 (3)	0.0056 (3)
Mn1	0.0250 (6)	0.0294 (7)	0.0240 (7)	0.000	−0.0007 (6)	0.000
N1	0.024 (2)	0.025 (2)	0.028 (3)	−0.002 (2)	−0.002 (2)	0.002 (2)
N2	0.028 (3)	0.036 (3)	0.028 (3)	0.003 (2)	−0.004 (2)	−0.007 (3)
N3	0.023 (2)	0.030 (2)	0.025 (3)	−0.0023 (19)	−0.004 (2)	0.002 (2)
N4	0.031 (3)	0.038 (3)	0.034 (3)	0.004 (2)	0.005 (2)	0.000 (3)
C1	0.030 (3)	0.032 (3)	0.025 (3)	0.000 (3)	0.002 (3)	−0.004 (3)
C2	0.024 (3)	0.035 (4)	0.038 (4)	−0.009 (3)	−0.001 (3)	−0.002 (3)
C3	0.030 (4)	0.041 (4)	0.028 (4)	0.004 (3)	−0.007 (3)	−0.005 (3)
C4	0.025 (3)	0.025 (3)	0.024 (3)	0.009 (3)	−0.002 (3)	−0.008 (2)
C5	0.034 (3)	0.030 (3)	0.033 (4)	−0.003 (3)	−0.003 (3)	0.004 (3)
C6	0.027 (3)	0.031 (3)	0.042 (4)	0.011 (3)	0.006 (3)	0.004 (3)
C7	0.037 (4)	0.039 (4)	0.036 (4)	0.006 (4)	0.003 (3)	−0.009 (3)
C8	0.033 (3)	0.021 (3)	0.022 (3)	−0.004 (3)	0.002 (3)	0.006 (2)
O1	0.070 (3)	0.066 (4)	0.077 (4)	−0.027 (3)	0.007 (3)	0.002 (3)
N5	0.046 (5)	0.033 (5)	0.032 (5)	0.000	0.000	−0.003 (4)
C9	0.032 (4)	0.065 (7)	0.040 (6)	0.000	0.000	−0.017 (6)

### Geometric parameters (Å, °)

**Table:**

Mn1—Br1	2.6140 (9)	C2—C3	1.386 (8)
Mn1—N1^i^	2.292 (5)	C2—H2	0.9500
Mn1—N1	2.292 (5)	C3—H3	0.9500
Mn1—N3	2.306 (5)	C4—C8^i^	1.505 (8)
Mn1—N3^i^	2.306 (5)	C5—C6	1.385 (8)
Mn1—Br1^i^	2.6140 (9)	C5—H5	0.9500
N1—C1	1.338 (6)	C6—C7	1.370 (8)
N1—C4	1.347 (7)	C6—H6	0.9500
N2—C3	1.336 (6)	C7—H7	0.9500
N2—C4	1.336 (7)	C8—C4^i^	1.505 (8)
N3—C5	1.338 (6)	O1—N5	1.217 (5)
N3—C8	1.344 (7)	N5—O1^ii^	1.217 (5)
N4—C8	1.326 (7)	N5—C9	1.466 (9)
N4—C7	1.334 (7)	C9—H9A	0.9800
C1—C2	1.379 (7)	C9—H9B	0.9800
C1—H1	0.9500	C9—H9C	0.9800
			
N1^i^—Mn1—N1	158.0 (2)	C3—C2—H2	121.3
N1^i^—Mn1—N3	71.06 (16)	N2—C3—C2	122.3 (6)
N1—Mn1—N3	93.09 (15)	N2—C3—H3	118.8
N1^i^—Mn1—N3^i^	93.09 (15)	C2—C3—H3	118.8
N1—Mn1—N3^i^	71.06 (16)	N2—C4—N1	127.2 (5)
N3—Mn1—N3^i^	89.5 (2)	N2—C4—C8^i^	117.8 (5)
N1^i^—Mn1—Br1	101.45 (11)	N1—C4—C8^i^	115.1 (5)
N1—Mn1—Br1	92.87 (11)	N3—C5—C6	121.6 (6)
N3—Mn1—Br1	88.07 (11)	N3—C5—H5	119.2
N3^i^—Mn1—Br1	163.59 (10)	C6—C5—H5	119.2
N1^i^—Mn1—Br1^i^	92.87 (11)	C7—C6—C5	116.8 (5)
N1—Mn1—Br1^i^	101.45 (11)	C7—C6—H6	121.6
N3—Mn1—Br1^i^	163.59 (10)	C5—C6—H6	121.6
N3^i^—Mn1—Br1^i^	88.07 (11)	N4—C7—C6	123.1 (6)
Br1—Mn1—Br1^i^	98.66 (5)	N4—C7—H7	118.4
C1—N1—C4	115.6 (5)	C6—C7—H7	118.4
C1—N1—Mn1	125.4 (4)	N4—C8—N3	126.3 (6)
C4—N1—Mn1	118.7 (4)	N4—C8—C4^i^	117.4 (5)
C3—N2—C4	115.4 (5)	N3—C8—C4^i^	116.2 (5)
C5—N3—C8	116.3 (5)	O1—N5—O1^ii^	121.6 (8)
C5—N3—Mn1	125.1 (4)	O1—N5—C9	119.2 (4)
C8—N3—Mn1	117.3 (4)	O1^ii^—N5—C9	119.2 (4)
C8—N4—C7	115.7 (5)	N5—C9—H9A	109.5
N1—C1—C2	122.0 (5)	N5—C9—H9B	109.5
N1—C1—H1	119.0	H9A—C9—H9B	109.5
C2—C1—H1	119.0	N5—C9—H9C	109.5
C1—C2—C3	117.4 (5)	H9A—C9—H9C	109.5
C1—C2—H2	121.3	H9B—C9—H9C	109.5
			
N1^i^—Mn1—N1—C1	130.5 (4)	Mn1—N1—C1—C2	175.5 (4)
N3—Mn1—N1—C1	87.8 (4)	N1—C1—C2—C3	−1.4 (8)
N3^i^—Mn1—N1—C1	176.2 (4)	C4—N2—C3—C2	0.8 (8)
Br1—Mn1—N1—C1	−0.4 (4)	C1—C2—C3—N2	0.8 (9)
Br1^i^—Mn1—N1—C1	−99.9 (4)	C3—N2—C4—N1	−2.1 (8)
N1^i^—Mn1—N1—C4	−54.4 (4)	C3—N2—C4—C8^i^	178.5 (5)
N3—Mn1—N1—C4	−97.1 (4)	C1—N1—C4—N2	1.6 (8)
N3^i^—Mn1—N1—C4	−8.7 (4)	Mn1—N1—C4—N2	−174.0 (4)
Br1—Mn1—N1—C4	174.7 (4)	C1—N1—C4—C8^i^	−179.0 (4)
Br1^i^—Mn1—N1—C4	75.3 (4)	Mn1—N1—C4—C8^i^	5.4 (6)
N1^i^—Mn1—N3—C5	178.2 (5)	C8—N3—C5—C6	0.5 (7)
N1—Mn1—N3—C5	−17.4 (4)	Mn1—N3—C5—C6	−166.5 (4)
N3^i^—Mn1—N3—C5	−88.4 (5)	N3—C5—C6—C7	0.3 (8)
Br1—Mn1—N3—C5	75.4 (4)	C8—N4—C7—C6	3.8 (9)
Br1^i^—Mn1—N3—C5	−169.8 (3)	C5—C6—C7—N4	−2.6 (9)
N1^i^—Mn1—N3—C8	11.3 (4)	C7—N4—C8—N3	−3.0 (9)
N1—Mn1—N3—C8	175.7 (4)	C7—N4—C8—C4^i^	178.6 (5)
N3^i^—Mn1—N3—C8	104.7 (4)	C5—N3—C8—N4	0.9 (8)
Br1—Mn1—N3—C8	−91.5 (4)	Mn1—N3—C8—N4	168.9 (4)
Br1^i^—Mn1—N3—C8	23.3 (7)	C5—N3—C8—C4^i^	179.3 (4)
C4—N1—C1—C2	0.3 (7)	Mn1—N3—C8—C4^i^	−12.7 (6)

Symmetry codes: (i) −*x*, *y*, −*z*+1/2; (ii) *x*, −*y*+1, −*z*.

### Hydrogen-bond geometry (Å, °)

**Table:**

*D*—H···*A*	*D*—H	H···*A*	*D*···*A*	*D*—H···*A*
C1—H1···Br1	0.95	2.87	3.542 (6)	129
C2—H2···Br1^iii^	0.95	2.91	3.802 (6)	156
C6—H6···Br1^iv^	0.95	2.86	3.752 (5)	157
C9—H9C···N2^v^	0.98	2.57	3.430 (5)	147

Symmetry codes: (iii) −*x*+1, *y*, −*z*+1/2; (iv) *x*+1/2, *y*−1/2, *z*; (v) −*x*+1/2, −*y*+1/2, *z*−1/2.
